# Conformational characterization of full-length X-chromosome-linked inhibitor of apoptosis protein (XIAP) through an integrated approach

**DOI:** 10.1107/S205225251901073X

**Published:** 2019-08-23

**Authors:** Panagis Polykretis, Enrico Luchinat, Alessio Bonucci, Andrea Giachetti, Melissa A. Graewert, Dmitri I. Svergun, Lucia Banci

**Affiliations:** a CERM – Magnetic Resonance Center, University of Florence, Via Luigi Sacconi 6, 50019 Sesto Fiorentino, Italy; b Department of Experimental and Clinical Biomedical Sciences ‘Mario Serio’, University of Florence, Viale Morgagni 50, 50134 Florence, Italy; c EMBL, Hamburg Outstation, European Molecular Biology Laboratory, Notkestrasse 85, 22607 Hamburg, Germany; d Department of Chemistry, University of Florence, Via della Lastruccia 3, 50019 Sesto Fiorentino, Italy

**Keywords:** X-chromosome-linked inhibitor of apoptosis protein, full-length XIAP, SAXS, EPR, integrative structural biology

## Abstract

The X-chromosome-linked inhibitor of apoptosis protein (XIAP) is a multidomain protein whose main function is to block apoptosis by caspase inhibition. XIAP is also involved in other signalling pathways, including NF-κB activation and copper homeostasis. XIAP is overexpressed in tumours, potentiating cell survival and resistance to chemotherapeutics, and has therefore become an important target for the treatment of malignancy. Despite the fact that the structure of each single domain is known, the conformation of the full-length protein has never been determined. Here, the first structural model of the full-length XIAP dimer, determined by an integrated approach using nuclear magnetic resonance, small-angle X-ray scattering and electron paramagnetic resonance data, is presented. It is shown that XIAP adopts a compact and relatively rigid conformation, implying that the spatial arrangement of its domains must be taken into account when studying the interactions with its physiological partners and in developing effective inhibitors.

## Abstract 2

The anti-apoptotic multidomain protein XIAP is shown to be a compact dimer in solution as determined by an integrative approach combining NMR, SAXS, EPR and molecular modelling.

## 1. Introduction   

The X-chromosome-linked inhibitor of apoptosis protein (XIAP) is a 497-residue cytoplasmic zinc-binding protein that is expressed in most human tissues (Liston *et al.*, 1996 ▸; The Human Protein Atlas; https://www.proteinatlas.org/). XIAP contains three zinc-binding baculovirus IAP repeat (BIR) domains in the N-terminal region, a ubiquitin-associated (UBA) domain and a C-terminal, zinc-binding Really Interesting New Gene (RING) domain (Mace *et al.*, 2010 ▸).

XIAP belongs to the IAP family, and was first recognized as a potent inhibitor of apoptosis, directly blocking the proteolytic activity of caspases (Deveraux *et al.*, 1997 ▸; Eckelman *et al.*, 2006 ▸). Specifically, XIAP binds and inhibits the effector caspases 3 and 7 through its BIR2 domain and a portion of the linker between BIR1 and BIR2 (Sun *et al.*, 1999 ▸; Riedl *et al.*, 2001 ▸; Chai *et al.*, 2001 ▸), while it inhibits the initiating caspase 9 through its BIR3 domain (Srinivasula *et al.*, 2001 ▸). XIAP is overexpressed in tumours, where it potentiates cell survival and resistance to chemotherapeutics owing to its anti-apoptotic activity. Thus, XIAP has become an important target for the development of cancer treatments aimed at antagonizing its interaction with caspases (Schimmer *et al.*, 2006 ▸; Nakagawa *et al.*, 2006 ▸; Mizutani *et al.*, 2007 ▸; Lopes *et al.*, 2007 ▸; Mannhold *et al.*, 2010 ▸; Fulda & Vucic, 2012 ▸; Baggio *et al.*, 2018 ▸).

XIAP is also involved in other important cellular processes. Through its BIR1 domain, XIAP is involved in the activation of the NF-κB transcription factor (Lu *et al.*, 2007 ▸). The C-terminal RING domain has E3 ubiquitin ligase activity (Nakatani *et al.*, 2013 ▸) that is responsible for the ubiquitin­ation of several substrates such as RIP1 and RIP2, which are involved in the pro-inflammatory TNF and NOD2 signalling pathways, respectively (Krieg *et al.*, 2009 ▸; Witt & Vucic, 2017 ▸; Goncharov *et al.*, 2018 ▸). Consequently, mutations in the *XIAP* gene have been related to inflammatory diseases such as X-linked lymphoproliferative syndrome type 2 (XLP2; Damgaard *et al.*, 2013 ▸) and inflammatory bowel disease (IBD; Pedersen *et al.*, 2014 ▸). Finally, the literature reports that XIAP plays an important role in maintenance of intracellular copper homeostasis and that its downregulation contributes to the onset of copper toxicosis, such as in Wilson’s disease (Mufti *et al.*, 2006 ▸; Galbán & Duckett, 2010 ▸).

The multifunctional roles of XIAP have raised several questions about how this protein is able to perform so many functions and which are the structural features that allow XIAP to engage in so many interactions. Indeed, all of these processes imply that XIAP constantly interacts with one or more different partners in the cell, and the spatial arrangement of its domains could differently modulate the various interactions. The structures of all of the single domains of XIAP have been characterized (Sun *et al.*, 1999 ▸; Liu *et al.*, 2000 ▸; Lu *et al.*, 2007 ▸; Tse *et al.*, 2011 ▸; Lukacs *et al.*, 2013 ▸; Nakatani *et al.*, 2013 ▸). XIAP contains five zinc fingers, one in each BIR domain and two in the C-terminal RING domain (Sun *et al.*, 1999 ▸; Liu *et al.*, 2000 ▸; Nakatani *et al.*, 2013 ▸; Hou *et al.*, 2017 ▸), and it has been demonstrated that the BIR1 and the RING domains form homodimers (Lu *et al.*, 2007 ▸; Nakatani *et al.*, 2013 ▸; Hou *et al.*, 2017 ▸). Despite the wealth of data available for the single domains, there is no information about their spatial arrangement, as the conformation of full-length XIAP has never been determined. Consequently, structural characterization of the full-length protein is essential in order to elucidate the relation between its structural features and the numerous cellular processes and interactions in which this protein is involved. In this study, we employed an integrative approach using data obtained by nuclear magnetic resonance (NMR), small-angle X-ray scattering (SAXS) and electron paramagnetic resonance (EPR) as energy restraints in *HADDOCK*. The obtained models provide the first insight into the spatial arrangement adopted by full-length XIAP in solution.

## 2. Materials and methods   

### 2.1. Protein expression and purification   

The gene encoding XIAP was cloned in the pENTR vector to use the Gateway cloning technology and was subcloned in the pDEST-HisMBP vector (which adds a His tag followed by maltose-binding protein at the N-terminus of the protein), utilizing the pENTR/TEV/D-TOPO cloning kit (Invitrogen). *Escherichia coli* BL21-CodonPlus (DE3)-RIPL cells were transformed with the plasmid pDEST-HisMBP-XIAP and grown in LB medium (or ^15^N M9 medium) supplemented with 100 µ*M* ZnSO_4_ at 37°C and 170 rev min^−1^. At mid-log phase, the cells were induced with 0.75 m*M* IPTG and then grown overnight at 18°C and 170 rev min^−1^. The cells were harvested and resuspended in 100 ml binding buffer (20 m*M* Tris, 1 m*M* TCEP, 5 m*M* imidazole pH 8) supplemented with protease-inhibitor tablets (Bayer) and lysed by sonication (10 s on and 50 s off at 60% amplitude for 40 min). The lysate was passed through a 5 ml HisTrap FF affinity column (GE Healthcare Life Sciences) and washed with elution buffer (20 m*M* Tris, 1 m*M* TCEP, 500 m*M* imidazole pH 8). The His-MBP tag was cleaved by overnight incubation with TEV with dialysis (5 l). As a final purification step, gel filtration using a HiLoad 16/600 Superdex 200 pg column (GE Healthcare Life Sciences) was performed in order to separate XIAP from the His-MBP tag and to transfer the protein into the final buffer (20 m*M* Tris, 0.5 m*M* TCEP pH 7.4). The eluted fractions were checked by SDS–PAGE (Supplementary Fig. S1) and those containing pure XIAP were collected and concentrated using an Amicon Ultra centrifugal filter device (50 kDa molecular-weight cutoff).

### 2.2. NMR   

NMR spectra were acquired at 310 K on a 700 MHz Bruker Avance Neo spectrometer equipped with a TCI CryoProbe. 2D ^1^H–^15^N HSQC data were obtained from a sample of ^15^N-XIAP (160 µ*M* monomer concentration) in 20 m*M* Tris buffer, 0.5 m*M* TCEP pH 7.4. The spectra were processed using *TopSpin* from Bruker.

### 2.3. SEC-MALS   

Size-exclusion chromatography combined with multi-angle light scattering (SEC-MALS) was performed on full-length XIAP (30 µ*M*) utilizing a Superdex 200 10/300 GL column (GE Healthcare Life Sciences) at a flow rate of 0.3 ml min^−1^. The instrumentation included multi-angle light scattering with a quasi-elastic light-scattering detector and a refractometer with extended range (Wyatt Technology) connected to a high-performance liquid-chromatography (HPLC) system.

### 2.4. CD   

Circular-dichroism experiments were performed on 2.2 µ*M* samples of full-length XIAP using a JASCO J-810 spectrometer. The spectra were processed with the JASCO *Spectra Manager* software suite by applying a nine-point smoothing function. Secondary-structure calculation was performed using the *BeStSel* (*Beta Structure Selection*) web tool (Micsonai *et al.*, 2018 ▸).

### 2.5. ICP-AES   

The zinc:protein ratio of full-length XIAP (5 µ*M*) was determined by inductively coupled plasma atomic emission spectrometry (ICP-AES) measurements carried out by a Varian 720 ES simultaneous ICP-AES equipped with a CETAC U5000 AT+ ultrasonic nebulizer.

### 2.6. Small-angle X-ray scattering   

Synchrotron-radiation X-ray scattering from full-length XIAP in solution was collected on the EMBL P12 beamline at the PETRA III storage ring, DESY, Hamburg, Germany (Blanchet *et al.*, 2015 ▸). Images were recorded using a photon-counting PILATUS 2M detector at a sample-to-detector distance of 3.1 m and a wavelength (λ) of 1.2 Å, covering the momentum-transfer range 0.01 < *s* < 0.5 Å^−1^, with *s* = 4πsinθ/λ, where 2θ is the scattering angle. To obtain data from a monodisperse sample, a size-exclusion chromatography column was directly coupled to the scattering experiment (SEC-SAXS). Here, the eluent from a Superdex 200 10/300 GL column (GE Healthcare Life Sciences) was passed through a UV cell (280 nm, Agilent) and then to the SAXS capillary, where 1 s sample exposures were recorded. 20 m*M* Tris, 0.5 m*M* TCEP pH 7.4 was used as the mobile phase for SEC. 100 µl of the purified sample (7.5 mg ml^−1^) was injected and the flow rate was 0.5 ml min^−1^. SAXS data were recorded from macromolecule-free fractions corresponding to the matched solvent blank (frames 1389–1804 s) which eluted directly after the peak corresponding to the separated XIAP dimers (elution time maximum = 20.7 min, 10.4 ml; frames 1244–1281 s). Data reduction to produce the final scattering profile of dimeric full-length XIAP was performed using standard methods. Briefly, 2D-to-1D radial averaging was performed using the *SASFLOW* pipeline (Franke *et al.*, 2017 ▸). *CHROMIXS* was used for the visualization and reduction of the SEC-SAXS data sets (Panjkovich & Svergun, 2018 ▸). Aided by the integrated prediction algorithms in *CHROMIXS*, the optimal frames within the elution peak and the buffer regions were selected. Single buffer frames were then subtracted from sample frames one by one, scaled and averaged to produce the final subtracted curve.

The indirect inverse Fourier transform of the SAXS data and the corresponding probable real-space scattering pair distance distribution [*p*(*r*) versus *r* profile] of full-length XIAP was calculated using *GNOM* (Svergun, 1992 ▸), from which the *R*_g_ and *D*_max_ were determined. The *p*(*r*) versus *r* profile was also used for volume and subsequent molecular-weight estimates of the XIAP dimers, as evaluated by the *DATPOROD* (Porod volume; Franke *et al.*, 2017 ▸), *DATMOW* (Fischer *et al.*, 2010 ▸) and *DATVC* (Rambo & Tainer, 2013 ▸) modules of the *ATSAS* 2.8 package. *Ab initio* bead modelling of XIAP was performed using ten independent runs of *DAMMIF* (Franke & Svergun, 2009 ▸); from this, the most probable model was selected for further analysis by *DAMAVER* (Volkov & Svergun, 2003 ▸). The *ab initio* modelling was performed with and without symmetry constraints (*P*2 symmetry to reflect the dimeric state of the protein). The resolution of the model ensemble was estimated with *SASRES* (Tuukkanen *et al.*, 2016 ▸). The *a priori* shape classification of the SAXS data was conducted with *DATCLASS* (Franke *et al.*, 2018 ▸). The molecular mass (MM) was evaluated based on concentration-independent methods as described in Hajizadeh *et al.* (2018 ▸).

The SAXS data (as summarized in Supplementary Table S2) and *ab initio* bead models, as well as the rigid-body reconstruction of full-length XIAP, have been deposited in the Small-Angle Scattering Biological Data Bank (SASBDB; Valentini *et al.*, 2015 ▸) under accession code SASDF24.

### 2.7. EPR   

In order to perform EPR and EPR-DEER experiments, the MTSL [*S*-(1-oxyl-2,2,5,5-tetramethyl-2,5-dihydro-1H-pyrrol-3-yl)methyl methanesulfonothioate] nitroxide spin label was selected for site-directed spin labelling (SDSL; Hubbell *et al.*, 2013 ▸; Klare, 2013 ▸). WT XIAP possesses four cysteine residues (Cys12, Cys202, Cys213 and Cys351) that are not involved in zinc coordination. Given the homodimeric nature of XIAP, labelling the same cysteine residue on each monomer would provide a symmetrical distance restraint. Mutations were sequentially introduced into the WT XIAP gene using the QuikChange II mutagenesis kit (Invitrogen), and the resulting genes were checked by DNA sequencing. Two triple mutants of XIAP were chosen for the SDSL reaction, termed XIAP C202 (*i.e.* XIAP C12A, C213G, C351S) and XIAP C351 (*i.e.* XIAP C12A, C202S, C213G).

The SDSL reaction was performed by incubating the XIAP mutants with a tenfold excess of MTSL. The reaction was kept for 2 h at room temperature under continuous agitation, after which an identical amount of MTSL was added to the solution to improve the labelling yield. After 4 h, unreacted spin label was eliminated by washing the reaction solution several times with 20 m*M* Tris pH 7.4 buffer using an Amicon Ultra centrifugal filter device (50 kDa molecular-weight cutoff). The resulting samples were checked by X-band EPR to ensure complete MTSL removal and to calculate the relative spin concentrations from double integration of the signals. Using a calibration curve, the total spin concentrations were 30 and 55 µ*M* for the two XIAP mutants labelled with MTSL nitroxide on Cys202 (XIAP C202R1, dimer concentration 15 µ*M*) and Cys351 (XIAP C351R1, dimer concentration 25 µ*M*), respectively, indicating an almost complete labelling reaction. X-band (9.8 GHz) continuous-wave EPR (CW-EPR) experiments were performed at room temperature on a Bruker ELEXSYS E580 spectrometer. The parameters used were as follows: microwave power = 10 mW, magnetic field modulation amplitude = 0.1 mT, field sweep = 10 mT, receiver gain = 60 dB. The spectra were accumulated nine times to increase the signal-to-noise ratio. Simulations were performed with the *SimLabel* program (a GUI for the *EasySpin* software; Etienne *et al.*, 2017 ▸) to obtain the components of the experimental spectra and the relative parameters (*g*-tensor, *A* splitting constants and τ_C_ correlation times).

Q-band experiments were performed on the same samples analysed using CW-EPR with the standard EN5107D2 resonator and an Oxford helium system to keep the temperature at 50 K. Four-pulse DEER experiments (34 GHz) were recorded with a Hahn-echo pulse sequence π/2–τ–π–τ–echo with π = 40 ns (π/2 = 20 ns) and τ_1_ = 200 ns; τ_2_ was set according to the relative spin–spin relaxation time. The pump ELDOR pulse was centred at the central resonance and the observed frequency was set with an offset of 56 MHz. The total acquisition time was 20–24 h to increase the signal-to-noise ratio. All DEER measurements were performed with an eight-step nuclear modulation average.

The *DeerAnalysis*2016 software was used to correct for background echo decay involving a second-order polynomial baseline correction, and successively to obtain the relative distance distribution (Jeschke, 2002 ▸, 2012 ▸). For the XIAP C202R1 sample, the fitted curve on the echo oscillation had an r.m.s. value of 0.017, while for the XIAP C351R1 sample the uncertainty could not be calculated owing to the absence of echo decay.

### 2.8. Ensemble analysis   

In *Ensemble Optimization Modelling* (*EOM*) analysis, ensembles of models with variable conformations are selected from a large pool of randomly generated models such that the scattering from the ensemble fits the experimental data, and the distributions of the overall parameters (*e.g.* *R*_g_ and *D*_max_) in the selected pool are compared with the original pool (Tria *et al.*, 2015 ▸). For the preliminary *EOM* analysis, 14 000 models with randomized linkers were generated based on the atomic structures from the individual domains: BIR1 (PDB entry 2poi; Lu *et al.*, 2007 ▸), BIR2 (PDB entry 4j3y; Lukacs *et al.*, 2013 ▸), BIR3 (PDB entry 4kmp; X. Li, J. Wang, S. M. Condon & Y. Shi, unpublished work), UBA (PDB entry 2kna; Hui *et al.*, 2010 ▸) and RING (PDB entry 4ic2; Nakatani *et al.*, 2013 ▸). The missing linker residues (217 in total) as well as 21 N-terminal residues make up ∼23% of the overall sequence. To account for the dimerization, *P*2 symmetry was partially applied by constraining the BIR1 as well as the RING dimer interfaces as seen in the crystal structure (PDB entries 2poi and 4ic2).

### 2.9. *HADDOCK* modelling   

The input model for *HADDOCK* (Dominguez *et al.*, 2003 ▸; van Zundert *et al.*, 2016 ▸) was generated starting from the atomic structures of the individual domains: BIR1 (PDB entry 2poi), BIR2 (PDB entry 1i3o; Riedl *et al.*, 2001 ▸), BIR3 (PDB entry 5m6e; Tamanini *et al.*, 2017 ▸), UBA (PDB entry 2kna) and RING (PDB entry 5o6t; Gabrielsen *et al.*, 2017 ▸). These structures were chosen to maximize the number of structurally defined segments. The domains were arranged in an extended, O-shaped configuration (Supplementary Fig. S3) using *PyMOL* v.1.4 (Schrödinger). The dimeric interfaces of BIR1 and RING were maintained as in the experimental structures. All of the unstructured residues were removed from the starting model and were subsequently reintroduced as extended loops, with the exception of the N-terminal 22 residues. In order to reconstruct the unstructured loops, an *XPLOR-NIH* (Schwieters *et al.*, 2003 ▸, 2006 ▸) protocol was used, in which the structured parts were kept fixed and the missing loop atoms were randomly generated. The structure was then minimized using a simulating-annealing protocol.

In the *HADDOCK* calculations the experimental restraints (*R*_g_ = 38 Å from SAXS and the inter-cysteine distance restraints Cys202–Cys202 = 38 ± 6 Å and Cys351–Cys351 > 70 Å from EPR-DEER) were set as unambiguous restraints. The *R*_g_ restraint weight was set to 0.01 in order to achieve a broader structure distribution. *C*2 symmetry distance restraints were also introduced. In order to preserve the BIR1 and RING homodimers, a set of unambiguous restraints between CA atoms on opposite sides of the interface were generated from the X-ray structures. In the first rigid-body phase of *HADDOCK* calculations all of the structured parts were fixed, which were then released in the final refinement. The calculations were performed at the local PBS cluster at CERM, Florence by calculating 40 000 structures in the rigid-body step and 4000 in the second step. The deviation from the experimental SAXS curve (χ^2^) was calculated for all of the 4000 resulting structures using *CRYSOL* (Svergun *et al.*, 1995 ▸).

## 3. Results and discussion   

**Figure 1:**
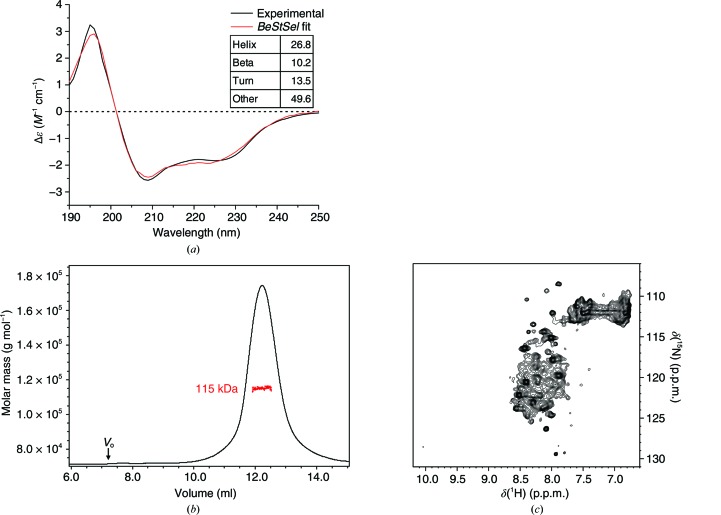
XIAP is a compact homodimer in solution. (*a*) Experimental CD spectrum (black) of full-length XIAP (2.2 µ*M*, 298 K) and *BeStSel* fitting (red). The secondary-structure content estimated from the fitting is shown. (*b*) SEC-MALS elution profile of full-length XIAP (30 µ*M*, 298 K). The refractive index is shown in black; the calculated molecular mass of the XIAP dimer peak is shown in red. (*c*) ^1^H–^15^N HSQC NMR spectrum of the full-length XIAP homodimer acquired at a dimer concentration of 80 µ*M* at 700 MHz and 310 K. ∼70 amide cross-peaks are detected in the central region of the spectrum, typical of unstructured proteins. No signals are detected from the folded domains owing to relaxation-induced broadening beyond detection.

**Figure 2:**
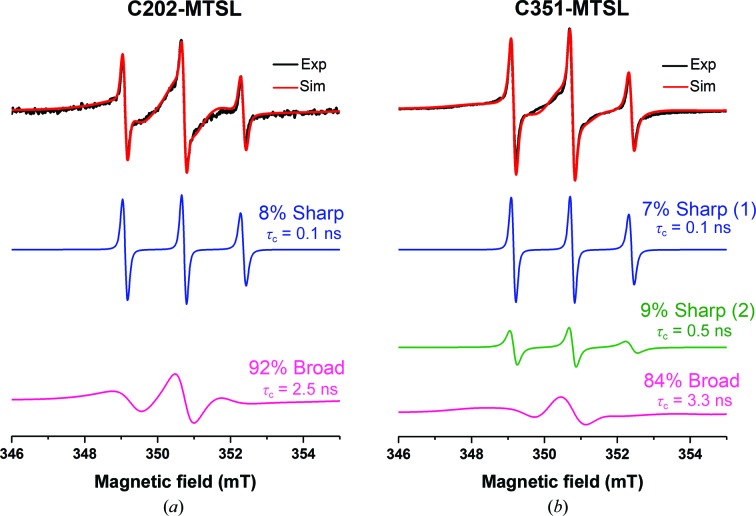
CW-EPR experiments on MTSL-labelled XIAP. X-band CW-EPR spectra at room temperature and relative simulations of (*a*) XIAP C202–MTSL and (*b*) XIAP C351–MTSL are shown. The experimental (blue) and simulated (red) spectra are overlaid. The sharp (blue/green) and broad (magenta) components of each simulated spectrum are shown below. The relative contribution and the calculated τ_C_ are indicated for each component.

The integrated approach used in this study involved the collection of several types of information for the structural characterization of full-length XIAP. The CD spectrum indicated that the purified protein is folded, and the secondary-structure content was estimated by fitting the CD data [Fig. 1 ▸(*a*)]. SEC-MALS experiments indicated that the protein is monodisperse in solution and forms a homodimer of approximately 115 kDa, consistent with the theoretical molecular mass of 113.4 kDa for the dimeric form [Fig. 1 ▸(*b*)]. The metallation state of the protein was determined by ICP-AES and resulted in ten zinc ions per XIAP homodimer, indicating that all of the binding sites are completely loaded with zinc. In principle, the multidomain nature of XIAP could allow a certain degree of relative flexibility of the single domains. Therefore, heteronuclear NMR spectroscopy was used to analyse the dynamic nature of the XIAP homodimer. Only a small number of amide cross-peaks (∼70) were visible in the central region of the ^1^H–^15^N HSQC spectrum of XIAP [Fig. 1 ▸(*c*)]. These signals are likely to arise from the unstructured regions of the protein, *i.e.* the N-terminus and the linkers between the domains. No signals typical of a folded protein were detected, indicating that XIAP is sufficiently rigid and reorients almost as a single dimeric form, thus slowing down the molecular tumbling and causing broadening beyond detection for the signals of the structured regions. NMR experiments optimized for high-molecular-weight systems did not provide additional signals, also owing to the low sample concentration imposed by the aggregation propensity of XIAP (data not shown). Further evidence of a rigid conformation assumed by the dimeric state of full-length XIAP was provided by EPR experiments. For this purpose, full-length XIAP was labelled with MTSL either at Cys202 on the BIR2 domain (XIAP C202R1) or at Cys351 on the BIR3 domain (XIAP C351R1). The X-band CW-EPR spectrum of XIAP C202R1 at room temperature exhibits a line shape typical of the presence of multi-motional components (Fig. 2 ▸, black line). Its simulation indicated that the overall signal is dominated by a broad component (τ_C_ = 2.5 ns), specific for a rigid structure, which accounts for 92% of the signal intensity [Fig. 2 ▸(*a*), pink line], while a minor sharp component (τ_C_ = 0.1 ns) typical of a very flexible conformation accounts for 8% of the signal [Fig. 2 ▸(*a*), blue line]. This indicates that XIAP essentially has a rigid structure around Cys202. Similar results were obtained for XIAP C351R1: the CW-EPR spectrum [Fig. 2 ▸(*b*), black line] shows the presence of two minor sharp components characteristic of very flexible conformations, with spin-label tumbling times of 0.1 and 0.5 ns [Fig. 2 ▸(*b*), pink and green lines, respectively], while most of the EPR signal (84%) arises from a broader component (τ_C_ = 3.3 ns) [Fig. 2 ▸(*b*), pink line], revealing that the protein also assumes a rigid structure in the proximity of Cys351. Overall, these findings confirm that the XIAP homodimer in solution adopts a relatively compact conformation.

**Figure 3:**
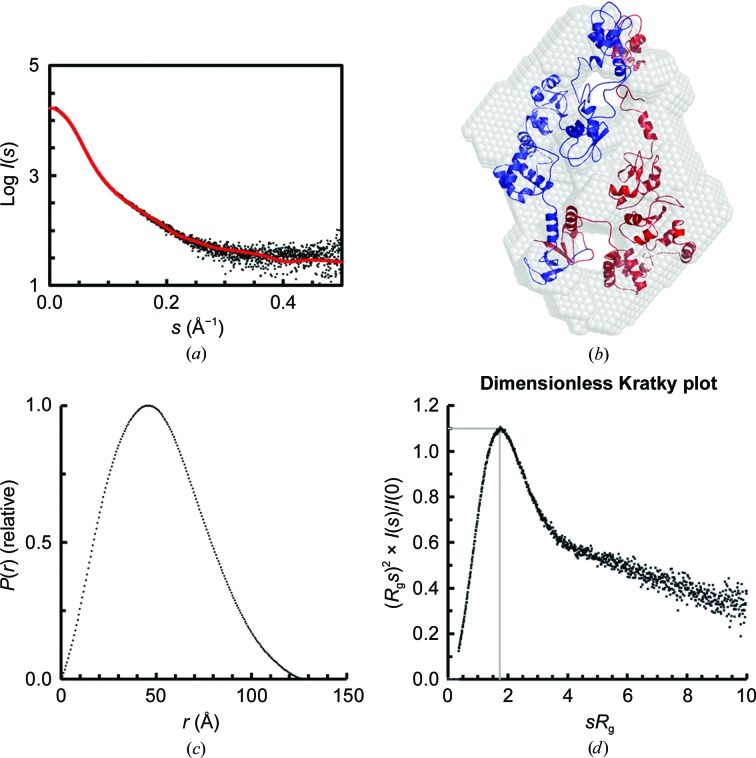
SAXS data, primary structural parameters and overall shape characteristics. (*a*) Averaged SEC-SAXS profile of XIAP through the dimeric elution peak (black) and the corresponding fit against the data from the best-fitting *HADDOCK* model (see Section 2; χ^2^ = 1.5 with no systematic deviation). (*b*) *Ab initio* bead-model reconstruction overlaid with the best-fitting *HADDOCK* model. The most probable *ab initio* model determined with *P*2 symmetry is shown. The resolution of the model was determined to be 34 ± 3 Å. (*c*) Distance distribution profile of XIAP. (*d*) Dimensionless Kratky plot. The typical peak position for globular proteins (*x* = 3^1/2^, *y* = 1.1) is indicated.

To further describe the conformation of XIAP, SAXS measurements were performed which provided the overall shape features of dimeric XIAP in solution. Trace amounts of higher molecular-weight species were removed during the chromatography step in the inline SEC-SAXS setup. In this way, the scattering intensity data were measured solely for the pure dimeric form. The radius of gyration (*R*_g_) through the dimer elution peak is consistent (36–38 Å), suggesting that the solute is indeed monodisperse. The final averaged SAXS profile of dimeric full-length XIAP is shown in Fig. 3 ▸(*a*). The Guinier plot of the SAXS data is linear, as expected for aggregate-free, monodisperse systems, yielding an *R*_g_ of 38 ± 0.6 Å. Compared with the expected molecular mass of 113.4 kDa, the mass calculated by SAXS (Table 1 ▸) suggests that XIAP has a relatively globular isotropic mass distribution. The *a priori* shape classification of the SAXS data places XIAP in the ‘flat/compact’ regime. The corresponding *p*(*r*) versus *r* profile [Fig. 3 ▸(*c*)] supports this observation as the distribution of vector lengths is almost Gaussian, with a maximum particle dimension (*D*_max_) of ∼130 Å. Correspondingly, the peak position in the dimensionless Kratky plot [Fig. 3 ▸(*d*)] shows a behaviour typical of a globular protein (Receveur-Brechot & Durand, 2012 ▸). The final structural parameters extracted from the data, including volume and molecular-mass estimates, are fully consistent with dimeric XIAP and with the SEC-MALS results (Table 1 ▸). In addition, the *ab initio* low-resolution structure indicated that the quaternary structure of the XIAP homodimer displays a disc-like flat conformation [Fig. 3 ▸(*b*)].

**Figure 4:**
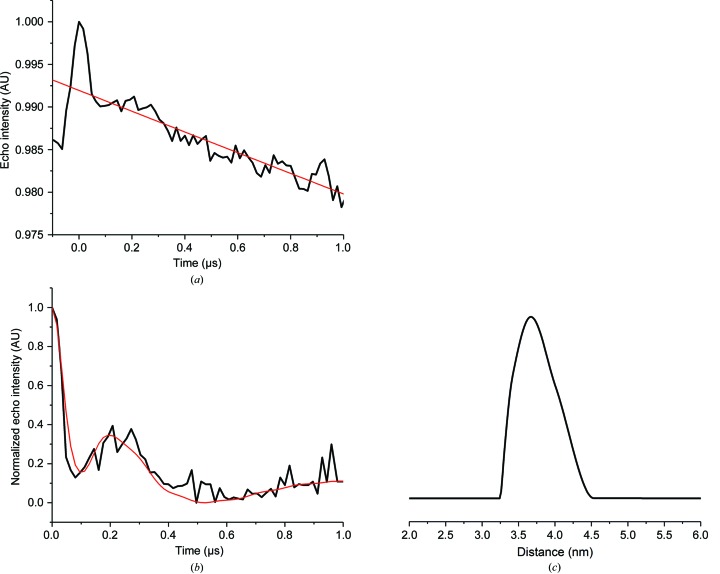
Q-band four-pulse DEER trace obtained for XIAP C202R1. (*a*) Experimental DEER trace (black) and estimated background (red). (*b*) DEER trace after background correction (black) and the relative fitting obtained by Tikhonov regularization (red). (*c*) Calculated distance distribution. The experimental data were treated using *DeerAnalysis*2016.

We further defined the relative position of the single domains in the XIAP homodimer by performing Q-band EPR-DEER measurements on XIAP C202R1 and XIAP C351R1. Since the protein is a homodimer, the values calculated from the DEER experiments correspond to the distances between two spin-labelled cysteines Cys202 (or Cys351) located one on each monomer. For C202R1, the modulation depth was estimated to be 0.012 and the calculated distance distribution had a single maximum at 38 ± 6 Å (Fig. 4 ▸), while for C351R1 the oscillation of the echo-detected signal was not observed (Supplementary Fig. S2), suggesting that the two Cys351 residues of the monomers are located farther than the estimated upper limit of the DEER experiment under our experimental conditions (>70 Å).

In order to obtain a family of models of the XIAP dimer that would satisfy the constraints derived from NMR (*i.e.* the absence of fast-tumbling domains) and EPR (*i.e.* the distances between cysteine pairs), while simultaneously minimizing the deviation from the SAXS curve, an integrated approach was devised. Specifically, *HADDOCK* calculations were first run to generate an ensemble of models that satisfy our experimental constraints, followed by a scoring based on the χ^2^ from the SAXS curve calculated with *CRYSOL* and a final selection of representative models by *EOM*. The initial input for *HADDOCK* was constructed as an O-shaped dimer (Supplementary Fig. S3), in which the two monomers are held together by the BIR1–BIR1 and RING–RING interfaces and the linkers are in an extended conformation, in order to facilitate the convergence towards shapes compatible with the flat discoid obtained from the *ab initio* modelling. The *R*_g_ from the SAXS data was used as a loose energy constraint in *HADDOCK*, while the inter-cysteine distances and the known dimer interfaces were employed as unambiguous distance restraints. Owing to the high degeneracy of the system, a large fraction of models of the resulting ensemble satisfied the experimental constraints (Supplementary Fig. S4). The ranking based solely on the deviation from the SAXS curve shows that a few individual models could adequately reproduce the curve, with the best-fitting model fitting the data with χ^2^ = 1.5 [Fig. 3 ▸(*b*)] and a total of six structures fitting the data with a χ^2^ below 2.1. Notably, the Cys351–Cys351 distance distribution in a subset of models that better reproduce the SAXS curve is shifted towards longer distances, peaking at around 70 Å [Supplementary Fig. S4(*b*)], therefore suggesting that the absence of modulation in the DEER experiment indeed results from a long distance instead of a broad distribution of shorter distances.

**Figure 5:**
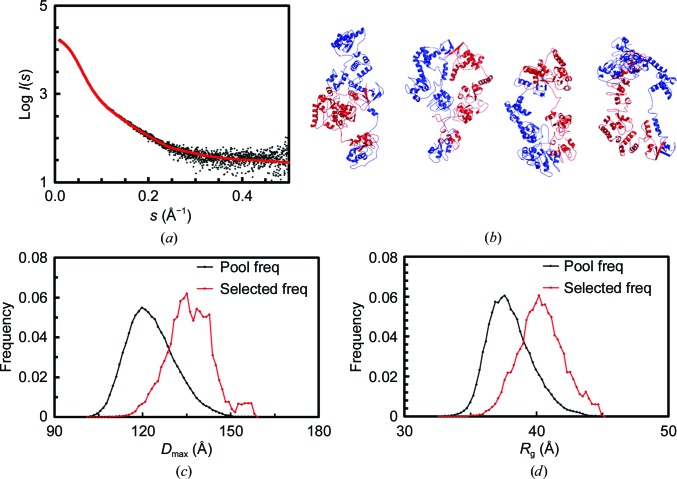
Flexibility assessment performed using *EOM* starting from the ensemble of *HADDOCK* models. (*a*) Fit (red) against the SAXS data (black) with the *EOM* approach (χ^2^ = 1.4 with no systematic deviation). (*b*) Representative models are shown as cartoons. The monomeric units are shown in red and blue, respectively. (*c*, *d*) Distribution for the structural parameters *D*_max_ (*c*) and *R*_g_ (*d*) of selected models (red) compared with those of the initial random pool (black).

Flexibility analysis performed using *EOM* on the *HADDOCK* ensemble further improved the fitting (χ^2^ = 1.4), yielding a family of representative models that satisfy all of the experimental constraints, being sufficiently compact to be consistent with the slow tumbling rate observed by NMR (Fig. 5 ▸ and Table 2 ▸). Notably, a similar flexibility analysis performed on randomly generated conformers without taking into account the DEER-derived distances resulted in a worse fit of the SAXS curve (χ^2^ = 1.9; Supplementary Fig. S5 and Table S1). It is also interesting that for random generation the overall sizes of the *EOM*-selected models were significantly smaller than the averages over the random pools [Supplementary Figs. S5(*c*) and 5(*d*)], whereas for the *HADDOCK* ensembles the selected models were generally more extended than the pool averages [Figs. 5 ▸(*c*) and 5 ▸(*d*)]. These results indicate that the integrated approach confined XIAP to a narrower conformational space, allowing a more thorough sampling of realistic conformers.

**Figure 6:**
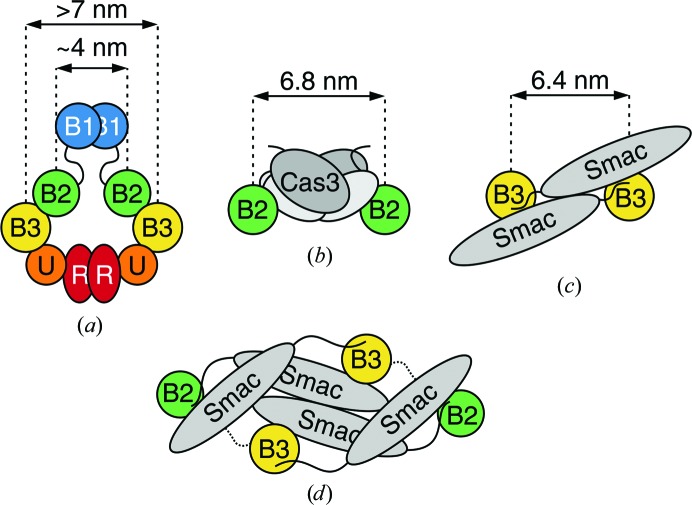
Schematic drawings showing the relative positions of the BIR2 and BIR3 domains and the distance between cysteines in the XIAP dimer and in complexes with different partners. (*a*) XIAP homodimer based on a representative model from the *HADDOCK* calculations performed in the present study. (*b*) Two BIR2 domains in complex with a caspase-3 dimer (PDB entry 1i3o; Riedl *et al.*, 2001 ▸). (*c*) Two BIR3 domains in complex with a Smac/DIABLO dimer (PDB entry 1g73; Wu *et al.*, 2000 ▸). (*d*) Two BIR2-BIR3 constructs in complex with a Smac/DIABLO tetramer based on a SAXS-derived model (Mastrangelo *et al.*, 2015 ▸). XIAP domains are labelled with the initial letter of each name; inter-cysteine distances are shown in (*a*), (*b*) and (*c*).

Taken together, from all of the evidence obtained XIAP is a compact oblate-shaped dimer in solution. This finding has important consequences when considering that XIAP has to interact with many partners to exert its functions (Fig. 6 ▸). In most cases such interactions take place between two copies of the involved domains of XIAP and the relative partners, as is the case for the BIR1–TAB (Lu *et al.*, 2007 ▸) and BIR2–caspase-3/7 complexes (Riedl *et al.*, 2001 ▸; Scott *et al.*, 2005 ▸), which are dimeric in the crystallographic structure, and the BIR2-BIR3–Smac/DIABLO complex, where it has been proposed that a Smac/DIABLO tetramer binds two BIR2-BIR3 pairs (Wu *et al.*, 2000 ▸; Huang *et al.*, 2003 ▸; Mastrangelo *et al.*, 2015 ▸). By comparing our model [Fig. 6 ▸(*a*)] with previous structural information, it can be observed that while the BIR1–TAB interaction is compatible with a compact XIAP dimer, the two BIR2 domains in complex with caspase-3 are further apart from each other [Cys202–Cys202 distance of 68.5 Å; Fig. 6 ▸(*b*)] than experimentally observed in the XIAP dimer (Cys202–Cys202 distance of 38 ± 6 Å), suggesting that the relative position of the BIR2 domains must change to allow binding to caspase-3/7. Likewise, the proposed arrangement of the two BIR2-BIR3 pairs bound to the Smac/DIABLO tetramer, although less clearly defined, conceivably involves a similar domain rearrangement [Figs. 6 ▸(*c*) and 6 ▸(*d*)].

In our view, the fact that the XIAP dimer must undergo a conformational rearrangement to allow binding to its partners is highly relevant when seeking to design more potent inhibitors of XIAP. Indeed, previous efforts to develop IAP inhibitors revealed that dimeric ligands showed higher potency with respect to their monomeric analogues (Hennessy *et al.*, 2013 ▸; LaCasse *et al.*, 2008 ▸; Lecis *et al.*, 2012 ▸). Our results provide a plausible explanation for the increased potency of these dimeric compounds, as the dimeric compact structure of XIAP could allow the compounds to bridge the two monomers, thus stabilizing the protein in the caspase-free state.

## 4. Conclusions   

Given the number of different pathways in which XIAP is involved, it becomes necessary to determine whether they can be affected or modulated by the three-dimensional organization/architecture of the protein. This is especially relevant as the BIR domains of XIAP are being closely studied as potential anticancer drug targets (Fulda & Vucic, 2012 ▸). As is often the case with multidomain proteins, the structures of the single domains of XIAP have been relied upon for drug screening and for investigating protein–protein interactions, while the overall protein conformation has not been accounted for. Here, by integrating complementary data from different structural and biophysical techniques, we provided a first low-resolution model of full-length XIAP and assessed the degree of flexibility of the protein. In solution, XIAP behaves homogenously and is present as a homodimer. Most strikingly, our data indicate that the XIAP homodimer is overall a rigid entity, despite the fact that the unstructured N-terminus and the inter-domain linkers make up more than 20% of the overall sequence. This is suggested both by the results of *ab initio* modelling and by the undetectability of the folded domains within the XIAP dimer by solution NMR on nondeuterated samples. The presence of a compact conformation is confirmed by the family of models obtained by integrating data from EPR-DEER and SAXS. Furthermore, only ∼70 NMR signals from unfolded regions were detected, *i.e.* about one half of the total expected unfolded residues, suggesting that some of the inter-domain linkers could actually adopt a defined, more rigid conformation within the overall three-dimensional structure. Modelling the XIAP dimer based on the integration of SAXS and EPR-DEER data was a challenging task owing to the data being sparse compared with the huge number of degrees of freedom of the system. Despite this, our findings highlight the fact that XIAP assumes quite a compact and rigid conformation and should not be treated using simplistic ‘beads-on-a-string’ models when studying the interactions with its many partners. Eventually, this notion must be taken into account in the development of the next generation of XIAP inhibitors.

## Supplementary Material

Supplementary tables and figures. DOI: 10.1107/S205225251901073X/lz5027sup1.pdf

SASBDB reference: X-chromosome-linked inhibitor of apoptosis protein, SASDF24

## Figures and Tables

**Table 1:** Structural parameters for XIAP from SAXS data

*R*_g_ (from Guinier plot) (Å)	38 ± 0.6
*R*_g_ [from *p*(*r*) versus *r*] (Å)	39 ± 0.6
*D*_max_ (Å)	128
*V*_p_ (from Porod volume) (nm^3^)	194
*V*_p_ (from *DAMMIF*) (nm^3^)	184
MM (from Porod volume) (kDa)	121
MM (from *DATMOW*) (kDa)	120
MM (from *V*_c_) (kDa)	118
MM (from *DAMMIF*) (kDa)	92

**Table 2:** Structural parameters of the models selected by *EOM* analysis of the *HADDOCK* ensemble

Models	*R*_g_ (Å)	*D*_max_ (Å)	Fraction	Cys202–Cys202 distance (Å)	Cys351–Cys351 distance (Å)
Model 1	40	144	45	43	102
Model 2	41	130	11	45	82
Model 3	41	135	33	42	77
Model 4	41	135	11	44	83
Ensemble	41	136			
