# Supplementary material for: Conformational characterization of full-length X-chromosome-linked inhibitor of apoptosis protein (XIAP) through an integrated approach
Source: IUCrJ. 2019 Aug 23;6(Pt 5):948–57. doi: 10.1107/S205225251901073X (PMC6760453; doi:10.1107/S205225251901073X)
Supplement: Supplementary file 1 [file m-06-00948-sup1.pdf]

IUCrJ

Volume 6 (2019)

Supporting information for article:

Conformational characterization of full-length X-chromosome-linked inhibitor of apoptosis protein (XIAP) through an integrated approach

Panagis Polykretis, Enrico Luchinat, Alessio Bonucci, Andrea Giachetti, Melissa A. Graewert, Dmitri I. Svergun and Lucia Banci

Table S1 Structural parameters of the models selected by EOM analysis on an ensemble of models generated by connecting the structures of each domain with randomized linkers.

Models	R_g	D_{max}	Fraction	C202-C202	C351-C351
Model 1	40	1136	46	125	54
Model 2	44	141	8	68	40
Model 3	44	140	38	39	72
Model 4	40	152	8	90	38
Final ensemble	42	139			

Table S2 Data collection and SAXS-derived parameters for full length XIAP.

Data collection parameters	
Radiation source	Petra III (DESY, Hamburg, Germany)
Beamline	EMBL P12
Detector	Pilatus 2M
Beam geometry (Å)	2 x 12
Wavelength (Å)	1.24
Sample-detector distance (m)	3.1
s range (nm^{-1})	0.002 -0.45
Collection mode	SEC-SAXS
column	Superdex 200 10/300 GL
Flow rate	0.5 mL/min
Mobile phase	Tris 20 mM, TCEP 0.5 mM, pH 7.4
Injection volume, concentration	100 µl, 7.5 mg/ml
Exposure time (s)	1 sec
Temperature (°C)	20
Overall parameters (*merged profiles*)	
R_g from Guinier approximation (Å)	38 ± 0.6
R_g from PDDF (Å)	39 ± 0.6

D_{MAX} (Å)	15 ± 1
Porod Volume, V_P (nm ^{3})	207 ± 15
DAMMIF excluded volume (nm ^{3})	184 ± 15
Molecular weight from V_P (kDa)	130 ± 15
Molecular weight from excluded volume (kDa)	92 ± 15
Molecular weight from DATMOV, (kDa)	120± 15
Molecular weight from volume of correlation (kDa)	118± 15
Molecular weight from sequence (dimer, kDa)	113
Software employed	
Primary data reduction	SASFLOW
Data processing	PRIMUS
Ab initio modelling	DAMMIF/DAMMIN
Validation and averaging	DAMAVER
Calculation and comparison of scattering data	Crysol
Ensemble modelling	EOM
SASBDB accession code	SASDF24

Temporary link https://www.sasbdb.org/data/SASDF24/irylq6f8tr/

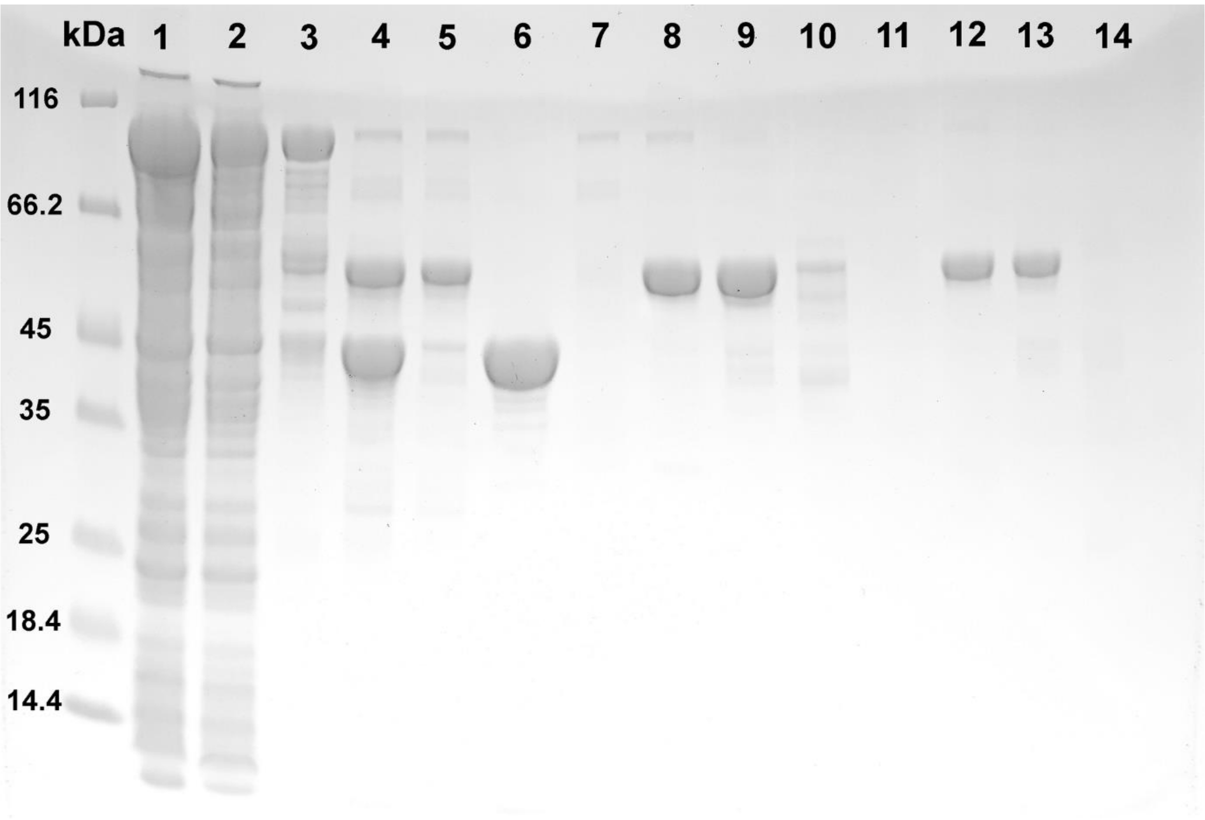

Figure S1 SDS-PAGE of the purification of XIAP: Lane 1) cell lysate; lane 2) cell lysate after HisTrap FF affinity chromatography; lane 3) elution of the HisTrap FF affinity chromatography; lane 4) protein sample after the incubation TEV protease; lane 6) flow-through of the HisTrap FF affinity chromatography after the incubation with TEV protease; lane 7) MBP eluted from the HisTrap FF column; lanes 7-10 & lanes 11-14) XIAP eluted from the HiLoad 16/600 Superdex 200 pg size exclusion chromatography column.

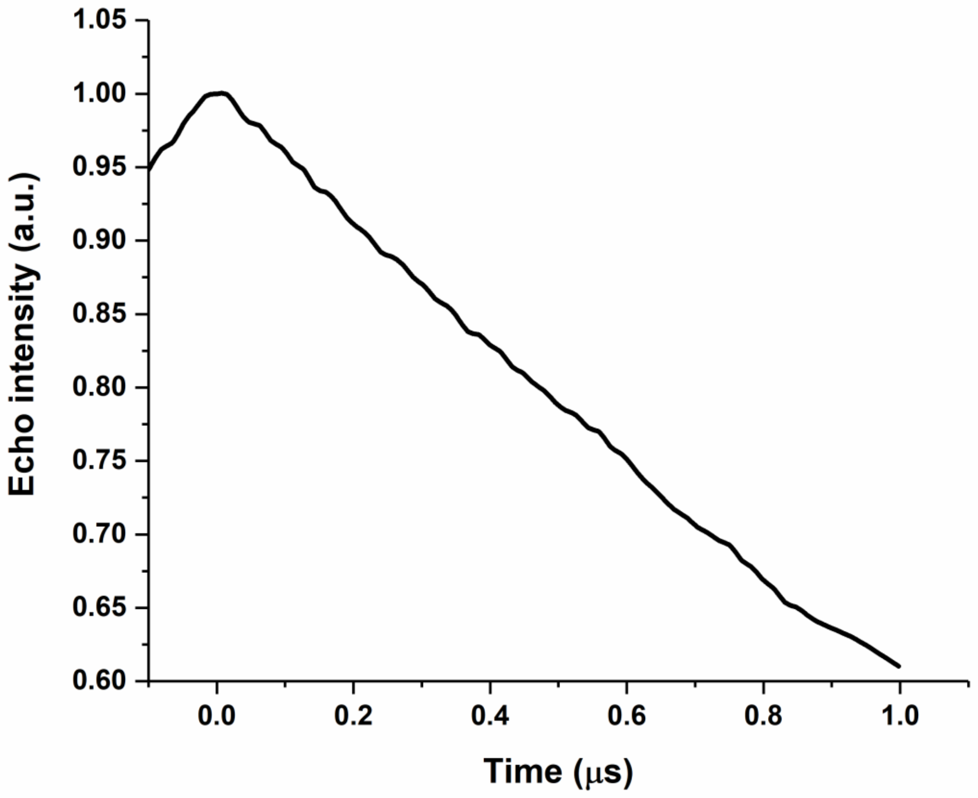

Figure S2 Q-band 4-pulse DEER trace obtained for XIAP C351R1. The absence of echo oscillation indicates a distance between the two MTSL spin labels of each monomer greater than the estimated upper limit of the DEER experiment (~70 Å in our experimental conditions).

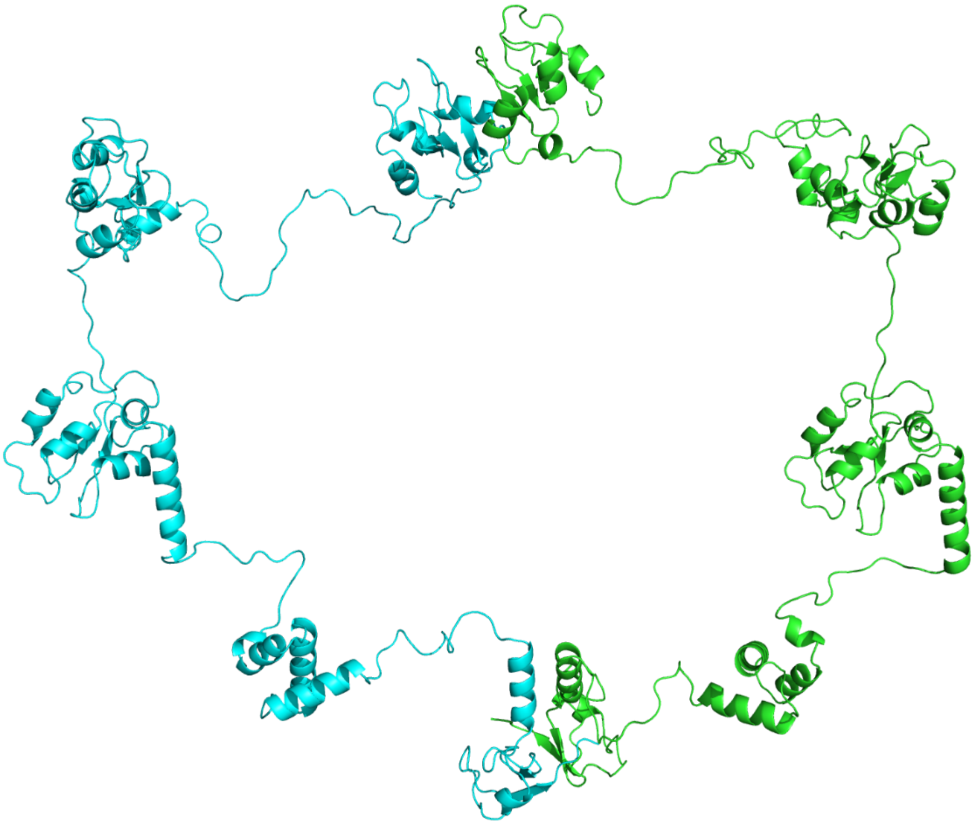

Figure S3 Initial input model of the XIAP dimer used for HADDOCK calculations. The two monomers are shown in cyan and green, respectively.

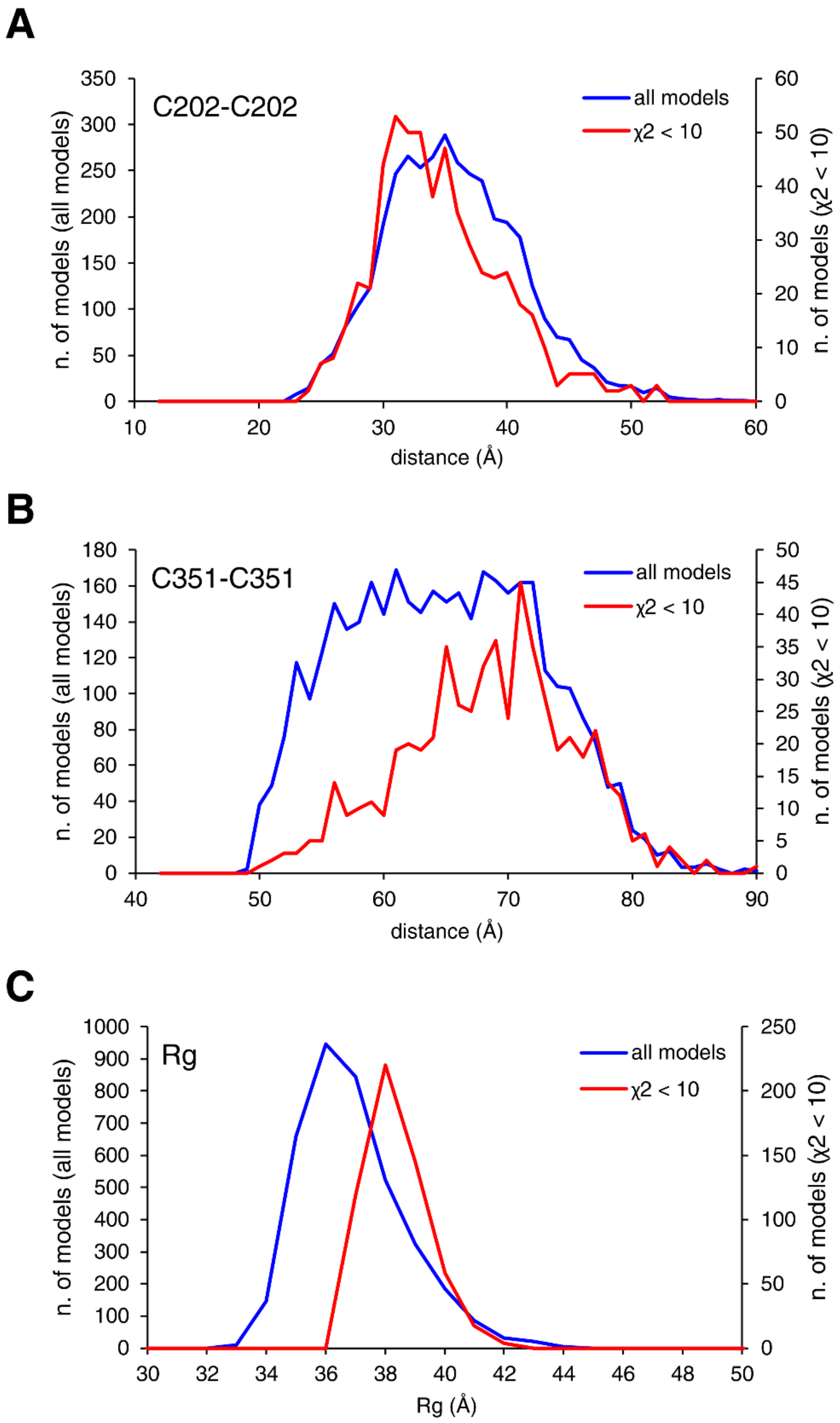

Figure S4 Distribution C202-C202 (A) and C351-351 (B) distances and Rg (C) calculated for all models obtained from HADDOCK (blue) compared to that calculated for a subset of models that better reproduce the SAXS curve (χ^2 < 10, red). The latter set matches more closely the distances measured by EPR-DEER.

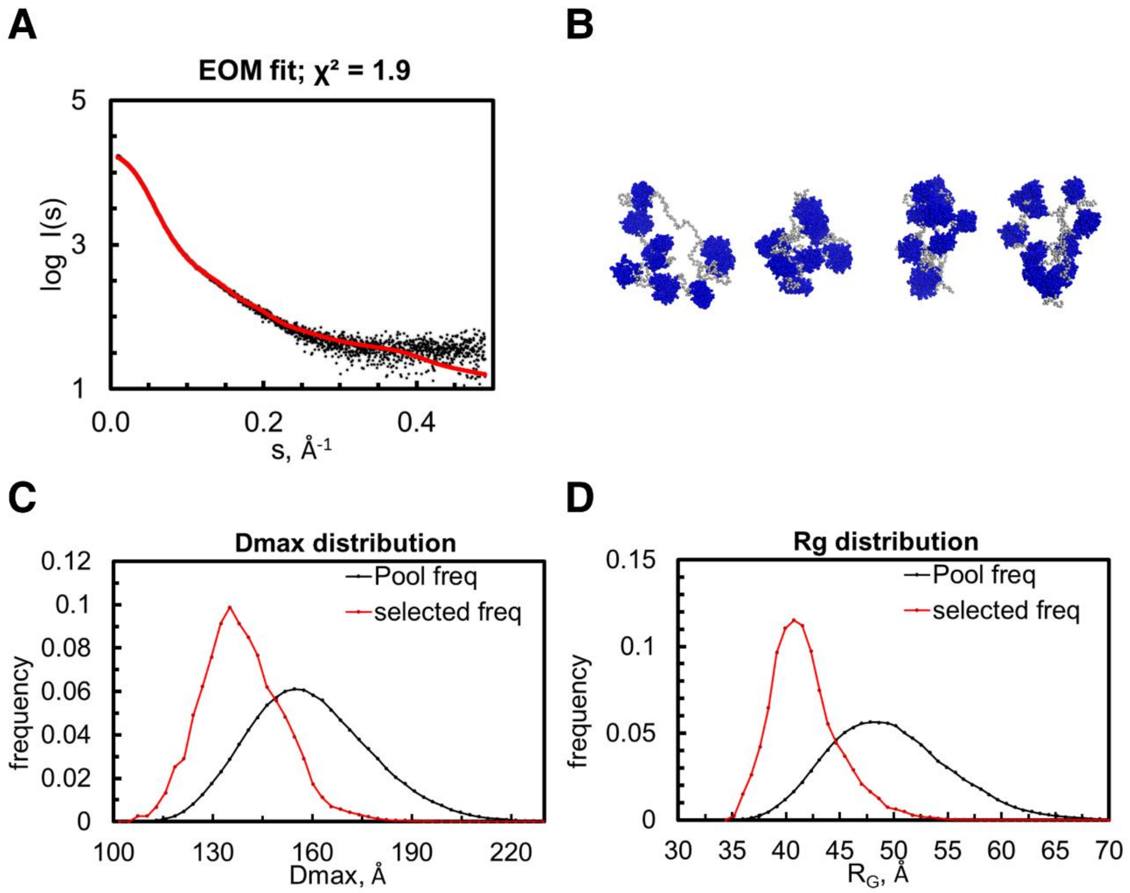

Figure S5 Flexibility assessment performed using EOM starting from an ensemble of models generated by connecting the structures of each domain with randomized linkers. A) Fit (red) against the SAXS data (black) with EOM (χ2=1.9 with little systematic deviations). B) representative models are shown as blue beads (folded domains) and grey beads represent linkers and N-terminal residues. C&D) Distributions of the structural parameters (C, Dmax; D, Rg,) of selected models (red) compared to those of initial random pool (black).
